# Association of CTLA-4 polymorphisms with hematologic malignancy susceptibility: a meta-analysis

**DOI:** 10.3389/fonc.2024.1467740

**Published:** 2024-10-11

**Authors:** Xuefen Yan, Nana Zhang, Gang Wang, Jiaheng Wang

**Affiliations:** Department of Hematology, the Quzhou Affiliated Hospital of Wenzhou Medical University, Quzhou People’s Hospital, Quzhou, China

**Keywords:** CTLA-4, hematologic malignancy, polymorphism, leukemia, meta-analysis

## Abstract

**Background:**

Recent studies have reported an association between Cytotoxic T-lymphocyte antigen-4 (CTLA-4) polymorphisms and hematologic malignancy susceptibility, while the results remain inconsistent. Hence, we performed a meta-analysis to investigate the association between CTLA-4 polymorphisms with hematologic malignancy susceptibility.

**Methods:**

A comprehensive and systematic search of Cochrane Library, PubMed, Embase databases was performed up to Sep. 20, 2024. The pooled odds ratio (OR) and its 95% confidence interval (CI) were used to determine the strength of the association between CTLA-4 polymorphisms and hematologic malignancy susceptibility. Statistical analysis was performed in STATA 12.0.

**Results:**

A total of 13 studies concerning the CTLA-4 49A/G, CTLA-4 60A/G, CTLA-4 318T/C, CTLA-4 1661A/G, and CTLA-4 319C/T polymorphisms were included in the meta-analysis. The pooled results suggested the CTLA-4 49A/G polymorphism was significantly associated with an increased hematologic malignancy risk (AA vs. GA+GG: OR = 1.77, 95% CI = 1.56-2.02), especially in NHL, multiple myeloma, and leukemia. Similarly, CTLA-4 319C/T polymorphism was found to be associated with decreased chronic lymphocytic leukemia risk. There was no significant association between the CTLA-4 60A/G, 318T/C, and 1661A/G polymorphism and hematologic malignancy risk.

**Conclusion:**

CTLA-4 49A/G and 319C/T polymorphisms were associated with hematologic malignancy susceptibility.

## Introduction

Hematologic malignancy originates from bone marrow and lymph nodes, and the heterogeneity of this disease drives the dysregulation of hematopoietic stem cells, leading to blockage and malignant proliferation of the hematopoietic differentiation system (1, 2). Malignant hematopoietic tumors are one of the most common cancers worldwide, and the incidence of these tumors has increased in recent years. According to global cancer statistics, approximately 176,200 people were diagnosed with hematologic malignancy in 2019 and more than 57,000 died of it (3, 4). Although the specific mechanisms of malignant hematopoietic tumors are not yet fully understood, increasing evidence suggests that genetic variations also play a role in influencing the risk of the disease, indicating that genetic factors play an important role in the development of hematologic malignancy.

The cytotoxic T-lymphocyte antigen-4 (CTLA-4) gene, encoding a transmembrane type 1 T-cell inhibitory receptor, plays a critical role as an immune checkpoint (5, 6). CTLA-4 is an important inhibitory factor for T-cell proliferation and activation by inducing Fas-independent cell apoptosis in activated T cells, which is crucial for regulating antitumor immune responses (7). Studies have shown that CTLA-4 inhibitors can block the binding of CTLA-4 to B7, inhibit the generation of T-cell inhibitory signals, and enhance specific antitumor immune responses (8). However, the CTLA-4 rs231775 genetic mutation can also affect the ability of CTLA-4 to bind to B7, thereby affecting T-cell activation (9–11). These results suggest that CTLA-4 genetic polymorphisms may be associated with the occurrence of cancer. The CTLA-4 gene is located on chromosome 2q33 and consists of 4 exons, which have multiple important single nucleotide polymorphisms (12). Recent studies have explored the relationship between CTLA-4 gene polymorphisms and susceptibility to hematologic malignancy (13–16). However, the results of different studies are contradictory. Considering the importance of CTLA-4 in tumor development, the purpose of this meta-analysis was to comprehensively analyze published studies to further clarify the relationship between CTLA-4 gene polymorphisms and hematologic malignancy.

## Methods

### Literature search

The study was performed according to the guidelines of the Preferred Reporting Items for Systematic Reviews and Meta-analyses statement (17). A comprehensive and systematic search of Cochrane Library, PubMed, Embase databases was performed up to Sep. 20, 2024. The following keywords we utilized: cytotoxic t-lymphocyte antigen 4, CTLA-4, polymorphism, variant, variation, mutation, SNP, leukemia, lymphoma, myeloma, and hematologic malignancy. There are no language restrictions on the search for articles. The references of the relevant potential articles on this topic were also manually searched to search for potentially relevant publications.

### Inclusion and exclusion criteria

Studies were included when met the following criteria: (1) case-control or cohort design in human subjects; (2) the associations between CTLA-4 gene polymorphisms and hematologic malignancy susceptibility; (3) completed data to calculate the odds ratio (OR) with the 95% confidence interval (95% CI); (4) control subjects satisfied Hardy–Weinberg equilibrium (HWE). The major exclusion criteria were: (1) duplicate publication; (2) non-human trials; and (3) lack of the full text or main genotyping data.

### Data extraction and quality assessment

Two investigators conducted literature screening, data extraction, and quality evaluation independently, and any discrepancies were resolved through consensus. The data extracted from the eligible articles included the first author, publication year, country, ethnicity, sample size, number of cases and controls for each genotype, P value of HWE, and score of quality assessment. The Newcastle-Ottawa scale (NOS) was adopted to assess the quality of included studies based on queue selection, comparability of queues, and evaluation of results.

### Statistical analysis

The statistical analysis was conducted using STATA 12.0 software. We used the χ² test to assess the HWE for each study. Pooled ORs and 95% CI were utilized to assess the association between the CTLA-4 polymorphisms and hematologic malignancy susceptibility in the dominant, recessive, homozygous, heterozygous, and allelic models. We assessed the heterogeneity between studies using the Cochrane Q-statistic test, and the unreliability was quantified using the I^2^ statistic. When there was significant heterogeneity (I^2^ > 50 or P < 0.05), the random-effects model was used, and otherwise, the fixed-effects model was used. Subgroup meta-analysis was performed by ethnicity and the cancer type. Sensitivity analysis was conducted to assess the stability of the results by omitting 1 study each time to exclude studies. The Begg funnel plots and Egger tests were used to assess the existence of publication bias. A 2-tailed P <.05 was considered significantly significant.

## Results

### Process of study selection and study characteristics

**Figure 1:**
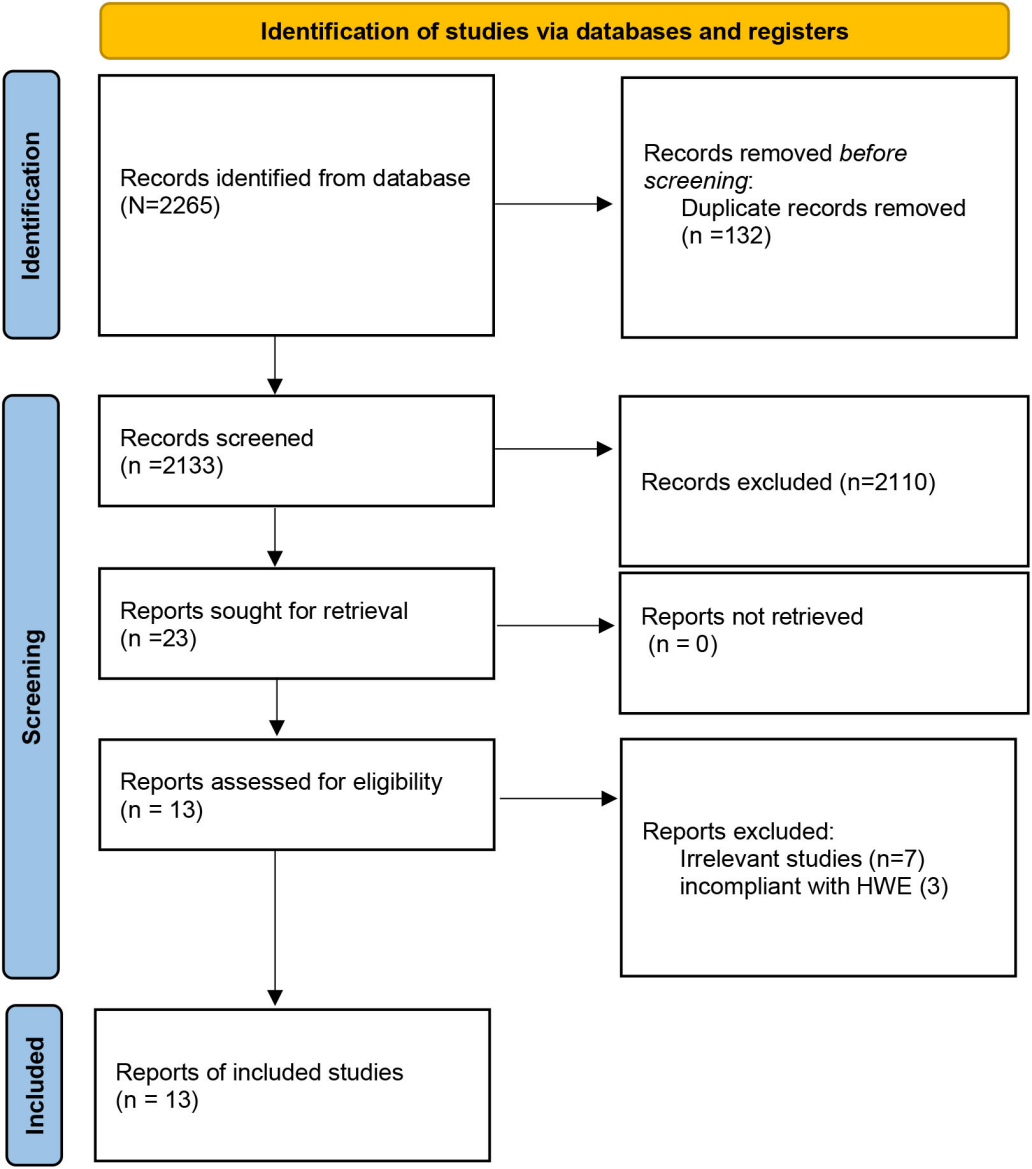
Flow chart of studies selection process.

**Figure 1** shows a study selection process. Through searching the Embase, PubMed, and Cochrane Library databases, 2265 studies were yielded from the database searches. After eliminating 132 duplicate articles, 2110 publications were further removed by screening titles and abstracts. After reading those articles, 13 articles were identified for this meta-analysis (13–16, 18–26), which included 12 studies for CTLA-4 49A/G (rs231775; located in exon 1 region), 5 studies for CTLA-4 60A/G (rs3087243; located in 3’-UTR region), 4 studies for CTLA-4 318T/C (rs5742909; located in promoter region), 4 studies for CTLA-4 1661A/G (rs4553808; located in promoter region), and 2 studies for CTLA-4 319C/T (rs5742909; located in promoter region) polymorphisms. The articles were published from 2004 to 2024, and the sample size ranged from 105 to 591. Among the included studies, 3 studies were from China, 2 studies from Italy, 2 studies from Poland, 2 studies from Egypt, and one study from Iran, Greece, Germany, Saudi Arabia, and Macedonia. The NOS score of all articles ranged from 6 to 8, implying that all included studies were of high quality. The selected study characteristics were summarized in **Table 1**.

**Table 1:** The main characteristics of the included studies.

First author	Year	Country	Ethnicity	Cancer type	Genotype methods	Sample size		Case			Controls			HWE (Control)	NOS score
						Case	Control	AA	AB	BB	AA	AB	BB		
CTLA-4 1661A/G (rs4553808)															
Cheng	2015	China	Asian	NHL	PCR-LDR	125	300	84	40	1	216	78	6	0.734	6
Liu	2013	China	Asian	NHL	PCR-LDR	291	300	220	66	5	216	78	6	0.734	6
Bonzheim	2008	Germany	Caucasian	NHL	PCR-RFLP	94	173	66	28	0	111	56	6	0.742	7
Ramzi	2020	Iran	Caucasian	Leukemia	PCR-RFLP	59	38	39	13	7	27	9	2	0.309	6
CTLA-4 318T/C (rs5742909)															
Cheng	2015	China	Asian	NHL	PCR-LDR	125	300	88	36	1	222	73	5	0.719	6
Liu	2013	China	Asian	NHL	PCR-LDR	291	300	222	64	5	222	73	5	0.719	6
Cheng	2006	China	Asian	NHL	PCR-RFLP	62	250	59	3	0	209	40	1	0.530	6
Bonzheim	2008	Germany	Caucasian	NHL	PCR-RFLP	94	173	78	13	3	140	29	4	0.107	7
CTLA-4 49A/G (rs231775)															
Cheng	2015	China	Asian	NHL	PCR-LDR	125	300	57	60	8	148	118	34	0.162	6
Khorshied	2014	Egypt	Caucasian	NHL	PCR-RFLP	181	200	72	88	21	106	76	18	0.415	6
Liu	2013	China	Asian	NHL	PCR-LDR	291	300	138	127	26	148	118	34	0.162	7
Cheng	2006	China	Asian	NHL	PCR-RFLP	62	250	34	26	2	119	102	29	0.323	6
Piras	2005	Italy	Caucasian	NHL	PCR-RFLP	100	128	74	23	3	74	43	11	0.199	6
Monne	2004	Italy	Caucasian	NHL	PCR-RFLP	44	76	36	7	1	38	32	6	0.837	6
Suwalska	2008	Poland	Caucasian	CLL	PCR-RFLP	170	224	56	84	30	71	106	47	0.523	8
Karabon	2012	Poland	Caucasian	Multiple Myeloma	PCR-RFLP	199	368	48	103	48	124	169	75	0.212	6
Velissari	2022	Greece	Caucasian	NHL	PCR-RFLP	146	60	57	69	20	37	17	6	0.078	7
Ramzi	2020	Iran	Caucasian	Leukemia	PCR-RFLP	59	38	14	42	3	16	19	3	0.413	6
Pavkovic	2003	Macedonia	Caucasian	CLL	PCR-RFLP	100	100	53	33	14	51	39	10	0.532	7
Alqahtani	2024	Saudi Arabia	Caucasian	Leukemia	TaqMan	98	128	60	34	4	58	62	8	0.105	8
CTLA-4 60A/G (rs3087243)															
Khorshied	2014	Egypt	Caucasian	NHL	PCR-RFLP	181	200	51	94	36	60	96	44	0.632	6
Liu	2013	China	Asian	NHL	PCR-LDR	291	300	197	84	10	208	82	10	0.586	7
Cheng	2006	China	Asian	NHL	PCR-RFLP	62	250	39	20	3	154	79	17	0.126	6
Cheng	2015	China	Asian	NHL	PCR-LDR	125	300	90	32	3	208	82	10	0.586	6
Bonzheim	2008	Germany	Caucasian	NHL	PCR-RFLP	94	173	24	41	29	35	95	43	0.185	7
CTLA-4 319C/T (rs5742909)															
Suwalska	2008	Poland	Caucasian	CLL	PCR-RFLP	170	333	121	42	7	267	62	4	0.851	8
Karabon	2012	Poland	Caucasian	Multiple Myeloma	PCR-RFLP	195	367	155	40	0	297	68	2	0.366	6

HWE, Hardy–Weinberg equilibrium; PCR-RFLP, polymerase chain reaction–restriction fragment length polymorphism; PCR-LDR, polymerase chain reaction–ligation detection reaction; NOS, Newcastle-Ottawa scale.

### Correlation between CTLA-4 49A/G polymorphism and hematologic malignancy risk

**Figure 2:**
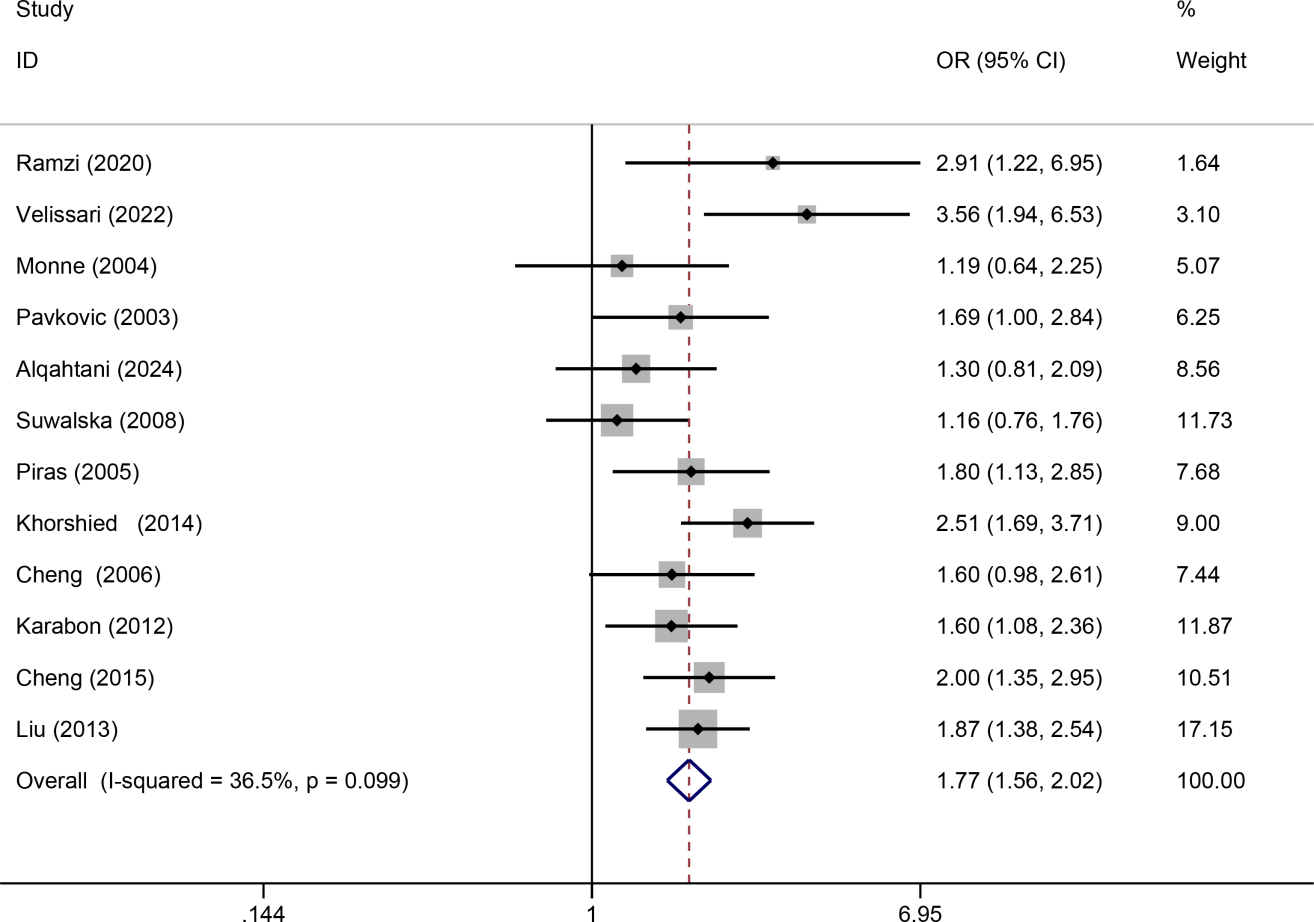
Forrest plot for association between CTLA-4 49A/G polymorphism and hematologic malignancy risk (AA vs. GA+GG).

Twelve relevant studies with 1575 cancer patients and 2172 controls were examined for the association between the CTLA-4 49A/G polymorphism and hematologic malignancy risk. The main results of the meta-analysis about CTLA-4 49A/G polymorphism were shown in **Table 2**. The pooled results indicated that CTLA-4 49A/G polymorphism was associated with hematologic malignancy risk under dominant model (AA vs. GA+GG: OR = 1.77, 95% CI = 1.56-2.02, P = 0.001) (**Figure 2**). There was no significant association with hematologic malignancy risk under the other four genetic models. However, in the stratification analysis by ethnicity, we observed that the Asian population was significantly related to an increased hematologic malignancy risk in the recessive model (GG vs. GA+AA: OR = 1.67, 95% CI = 1.10-2.53, P = 0.017), and dominant model (AA vs. GA+GG: OR = 1.85, 95% CI = 1.49-2.30, P = 0.001). The Caucasian population was significantly related to an increased hematologic malignancy risk in dominant model (AA vs. GA+GG: OR = 1.73, 95% CI = 1.47-2.04, P = 0.001). We then performed the subgroup analyses stratified by cancer types. The pooled ORs suggested that the CTLA-4 49A/G polymorphism was significantly associated with NHL risk (AA vs. GA+GG: OR = 1.98, 95% CI = 1.68-2.32, P = 0.001), multiple myeloma risk (AA vs. GA+GG: OR = 1.60, 95% CI = 1.08-2.36, P = 0.018; GG vs. AA: OR = 0.60, 95% CI = 0.37-0.99, P = 0.045; GA vs. AA: OR = 0.64, 95% CI = 0.42-0.96, P = 0.031; G vs. A: OR = 0.76, 95% CI = 0.60-0.98, P = 0.032), and leukemia risk (AA vs. GA+GG: OR = 2.91, 95% CI = 1.22-6.95, P = 0.016).

**Table 2:** Summary of meta-analysis of association of CTLA-4 49A/G (rs231775) polymorphism and hematologic malignancy risk.

Comparison		Studies	Overall effect			Heterogeneity	
			OR (95% CI)	*Z*-score	*p*-value	*I* ^2^ (%)	*p*-value
GG vs. GA+AA	Overall	12	1.13(0.92,1.39)	1.16	0.246	25.8	0.191
	Caucasian	9	0.98(0.77,1.25)	0.14	0.890	7.9	0.331
	Asian	3	1.67(1.10,2.53)	2.39	**0.017**	9.6	0.331
	NHL	7	1.36(1.00,1.85)	1.95	0.052	38.7	0.134
	CLL Multiple Myeloma	2 1	1.06(0.69,1.64) 0.81(0.53,1.22)	0.27 1.03	0.787 0.303	26.5 -	0.244 -
	Leukemia	2	1.58(0.59,4.24)	0.90	0.336	0	0.984
AA vs. GA+GG	Overall	12	1.77(1.56,2.02)	8.70	**0.001**	36.5	0.099
	Caucasian	9	1.73(1.47,2.04)	6.68	**0.001**	51.8	0.034
	Asian	3	1.85(1.49,2.30)	5.60	**0.001**	0	0.784
	NHL	7	1.98(1.68,2.32)	8.23	**0.001**	28.9	0.208
	CLL	2	1.34(0.97,1.86)	1.78	0.075	17.6	0.270
	Multiple Myeloma	1	1.60(1.08,2.36)	2.36	**0.018**	–	–
	Leukemia	2	1.56(1.03,2.36)	2.09	**0.037**	60.7	0.111
GG vs. AA	Overall	12	1.11(0.77,1.61)	0.54	0.586	51.3	0.020
	Caucasian	9	0.95(0.62,1.47)	0.23	0.817	49.3	0.046
	Asian	3	1.54(0.92,2.58)	1.63	0.103	17.2	0.299
	NHL	7	1.35(0.74,2.47)	0.99	0.323	61.7	0.016
	CLL	2	1.06(0.66,1.72)	0.25	0.800	0	0.349
	Multiple Myeloma	1	0.60(0.37,0.99)	2.00	**0.045**	–	–
	Leukemia	2	1.55(0.56,4.29)	0.84	0.402	0	0.434
GA vs AA	Overall	12	0.95(0.70,1.28)	0.34	0.736	73.8	0.001
	Caucasian	9	0.98(0.64,1.51)	0.08	0.935	80.4	0.001
	Asian	3	0.87(0.68,1.11)	1.12	0.263	0	0.567
	NHL	7	0.97(0.63,1.48)	0.14	0.886	78.4	0.001
	CLL	2	1.07(0.75,1.54)	0.39	0.700	0	0.584
	Multiple Myeloma	1	0.64(0.42,0.96)	2.15	**0.031**	–	–
	Leukemia	2	0.90(0.20,4.15)	0.13	0.894	88.1	0.004
G vs. A	Overall	12	1.06(0.85,1.32)	0.54	0.589	75	0.001
	Caucasian	9	1.05 (0.78,1.42)	0.32	0.750	80.3	0.001
	Asian	3	1.08 (0.90,1.31)	0.83	0.407	8.4	0.335
	NHL	7	1.14 (0.81,1.62)	0.76	0.449	81.9	0.001
	CLL	2	1.05 (0.83,1.33)	0.41	0.679	0	0.591
	Multiple Myeloma	1	0.76 (0.60,0.98)	2.15	**0.032**	–	–
	Leukemia	2	0.71 (0.39,1.31)	1.09	0.275	–	–

OR, odds ratio; CI, confidence interval, NHL, non-Hodgkin’s lymphoma; CLL, chronic lymphocytic leukemia. Bold type represents significantly difference.

### Correlation between CTLA-4 60A/G polymorphism and hematologic malignancy risk

Five relevant studies with 753 cancer patients and 1223 controls were examined for the association between the CTLA-4 60A/G polymorphism and hematologic malignancy risk. The overall analyses of 5 genetic models did not have any significant correlation between the CTLA-4 60A/G polymorphism and hematologic malignancy risk. As shown in **Table 3**, no association between CTLA-4 60A/G polymorphism and hematologic malignancy risk was detected in the ethnicity and cancer types subgroup analyses.

**Table 3:** Summary of meta-analysis of association of CTLA-4 60A/G (rs3087243) polymorphism and hematologic malignancy risk.

Comparison		Studies	Overall effect			Heterogeneity	
			OR (95% CI)	*Z*-score	*p*-value	*I* ^2^ (%)	*p*-value
GG vs. GA+AA	Overall	5	1.00(0.73,1.37)	0.001	1.000	0	0.747
	Caucasian	2	0.94(0.65,1.37)	0.31	0.756	20.6	0.262
	Asian	3	1.18(0.63,2.21)	0.51	0.607	0	0.841
	NHL	5	1.00(0.73,1.37)	0.001	1.000	0	0.747
AA vs. GA+GG	Overall	5	0.98(0.80,1.20)	0.22	0.829	0	0.806
	Caucasian	2	0.95(0.67,1.36)	0.27	0.786	6	0.302
	Asian	3	0.99(0.77,1.27)	0.07	0.941	0	0.774
	NHL	5	0.98(0.80,1.20)	0.22	0.829	0	0.806
GG vs. AA	Overall	5	1.08(0.75,1.55)	0.40	0.692	0	0.973
	Caucasian	2	1.03(0.66,1.61)	0.13	0.897	0	0.963
	Asian	3	1.17(0.62,2.21)	0.50	0.620	0	0.815
	NHL	5	1.08(0.75,1.55)	0.40	0.692	0	0.973
GA vs AA	Overall	5	1.02(0.82,1.26)	0.14	0.886	0	0.605
	Caucasian	2	1.07(0.74,1.56)	0.36	0.720	55.5	0.134
	Asian	3	0.99(0.76,1.28)	0.07	0.942	0	0.837
	NHL	5	1.02(0.82,1.26)	0.14	0.886	0	0.605
G vs. A	Overall	5	1.01(0.87,1.18)	0.16	0.870	0	0.951
	Caucasian	2	1.00(0.80,1.25)	0.02	0.986	0	0.932
	Asian	3	1.03(0.83,1.27)	0.24	0.807	0	0.716
	NHL	5	1.01(0.87,1.18)	0.16	0.870	0	0.951

OR, odds ratio; CI, confidence interval; NHL, non-Hodgkin’s lymphoma; CLL, chronic lymphocytic leukemia.

### Correlation between CTLA-4 318T/C polymorphism and hematologic malignancy risk

Four relevant studies with 572 cancer patients and 1023 controls were examined for the association between the CTLA-4 318T/C polymorphism and hematologic malignancy risk. The pooled results indicated that CTLA-4 318T/C was not significantly related to hematologic malignancy risk in all models. Then in the stratification analysis by ethnicity and cancer types, no significant association between CTLA-4 318T/C polymorphism and hematologic malignancy risk was discovered (**Table ​4**).

**Table 4:** Summary of meta-analysis of association of CTLA-4 318T/C (rs5742909) polymorphism and hematologic malignancy risk.

Comparison		Studies	Overall effect			Heterogeneity	
			OR (95% CI)	*Z*-score	*p*-value	*I* ^2^ (%)	*p*-value
CC vs. TC+TT	Overall	4	1.01(0.44,2.33)	0.02	0.982	0	0.879
	Caucasian	1	0.72(0.16,3.28)	0.43	0.669	–	–
	Asian	3	1.16(0.43,3.18)	0.30	0.766	0	0.802
	NHL	4	1.01(0.44,2.33)	0.02	0.982	0	0.879
TT vs. TC+CC	Overall	4	0.89(0.69,1.14)	0.93	0.352	46.5	0.132
	Caucasian	1	0.87(0.45,1.68)	0.41	0.679	–	–
	Asian	3	0.89(0.67,1.17)	0.84	0.403	64.2	0.061
	NHL	4	0.89(0.69,1.14)	0.93	0.352	46.5	0.132
CC vs. TT	Overall	4	1.03(0.44,2.38)	0.07	0.948	0	0.909
	Caucasian	1	0.74(0.16,3.40)	0.38	0.702	–	–
	Asian	3	1.18(0.43,3.23)	0.32	0.749	0	0.849
	NHL	4	1.03(0.44,2.38)	0.07	0.948	0	0.909
TC vs TT	Overall	4	1.13(0.87,1.47)	0.93	0.354	49.3	0.116
	Caucasian	1	1.24(0.61,2.53)	0.60	0.549	–	–
	Asian	3	1.12(0.84,1.48)	0.76	0.449	65.4	0.056
	NHL	4	1.13(0.87,1.47)	0.93	0.354	49.3	0.116
C vs. T	Overall	4	1.11(0.88,1.40)	0.88	0.377	39.8	0.173
	Caucasian	1	1.07(0.59,1.91)	0.21	0.832	–	–
	Asian	3	1.12(0.87,1.44)	0.87	0.384	60	0.082
	NHL	4	1.11(0.88,1.40)	0.88	0.377	39.8	0.173

OR, odds ratio; CI, confidence interval; NHL, non-Hodgkin’s lymphoma.

### Correlation between CTLA-4 1661A/G polymorphism and hematologic malignancy risk

Four relevant studies with 569 cancer patients and 811 controls were examined for the association between the CTLA-4 1661A/G polymorphism and hematologic malignancy risk. The pooled results indicated that no significant association between CTLA-4 1661A/G polymorphism and hematologic malignancy risk was found in overall and subgroup analyses. **Table 5** summarizes the evaluation results of the association between CTLA-4 1661A/G polymorphism and hematologic malignancy risk.

**Table 5:** Summary of meta-analysis of association of CTLA-4 1661A/G (rs4553808) polymorphism and hematologic malignancy risk.

Comparison		Studies	Overall effect			Heterogeneity	
			OR (95% CI)	*Z*-score	*p*-value	*I* ^2^ (%)	*p*-value
GG vs. GA+AA	Overall	4	1.32(0.62,2.78)	0.72	0.473	19.1	0.295
	Caucasian	2	1.15(0.38,3.51)	0.25	0.801	67.8	0.078
	Asian	2	1.46(0.53,4.06)	0.73	0.464	0	0.532
	NHL	3	1.99(0.78,5.04)	1.45	0.148	0	0.451
	Leukemia	1	0.41(0.08,2.10)	1.07	0.287	–	–
AA vs. GA+GG	Overall	4	0.95(0.74,1.21)	0.45	0.655	1.2	0.386
	Caucasian	2	0.87(0.55,1.38)	0.59	0.555	0	0.339
	Asian	2	0.98(0.74,1.30)	0.16	0.875	48.6	0.163
	NHL	3	0.93(0.72,1.19)	0.61	0.541	23.3	0.271
	Leukemia	1	1.26(0.52,3.05)	0.51	0.610	–	–
GG vs. AA	Overall	4	1.34(0.63,2.86)	0.76	0.446	18.1	0.300
	Caucasian	2	1.20(0.39,3.67)	0.31	0.753	68.8	0.073
	Asian	2	1.47(0.53,4.12)	0.74	0.460	0	0.602
	NHL	3	2.03(0.80,5.16)	1.48	0.139	0	0.467
	Leukemia	1	0.41(0.08,2.14)	1.05	0.292	–	–
GA vs AA	Overall	4	1.04(0.81,1.33)	0.29	0.774	0	0.449
	Caucasian	2	1.14(0.71,1.84)	0.55	0.586	0	0.762
	Asian	2	1.00(0.75,1.34)	0.001	0.999	57.1	0.127
	NHL	3	1.04(0.80,1.34)	0.30	0.767	24.3	0.267
	Leukemia	1	1.00(0.38,2.67)	0.001	1.000	–	–
G vs. A	Overall	4	1.06(0.86,1.32)	0.57	0.568	24.7	0.263
	Caucasian	2	1.13(0.76,1.68)	0.62	0.532	58.7	0.120
	Asian	2	1.04(0.80,1.34)	0.28	0.783	29.6	0.233
	NHL	3	1.11(0.89,1.39)	0.90	0.368	22.9	0.273
	Leukemia	1	0.70(0.33,1.45)	0.97	0.333	–	–

OR, odds ratio; CI, confidence interval; NHL, non-Hodgkin’s lymphoma.

### Correlation between CTLA-4 319C/T polymorphism and hematologic malignancy risk

Two relevant studies with 365 cancer patients and 700 controls were examined for the association between the CTLA-4 319C/T polymorphism and hematologic malignancy risk. Overall, CTLA-4 319C/T was not associated with hematologic malignancy susceptibility in all genetic models (**Table 6**). When subgroup analysis was conducted according to ethnicity, no significant association between CTLA-4 319 C/T polymorphism and hematologic malignancy risk was discovered. Using cancer types subgroup analyses, we observed that CTLA-4 319C/T polymorphism was significantly associated with the CLL risk in the recessive model (TT vs. TC+CC: OR: 0.28, 95%CI:0.08-0.98, P = 0.047), dominant model (CC vs. TC+TT: OR: 1.64, 95%CI:1.07-2.51, P = 0.024), homozygous model (TT vs. CC: OR: 0.26, 95%CI:0.07-0.90, P = 0.034), and allelic model (T vs C: OR: 0.60, 95%CI:0.42-0.87, P = 0.007).

**Table 6:** Summary of meta-analysis of association of CTLA-4 319C/T (rs5742909) polymorphism and hematologic malignancy risk.

Comparison		Studies	Overall effect			Heterogeneity	
			OR (95% CI)	*Z*-score	*p*-value	*I* ^2^ (%)	*p*-value
TT vs. TC+CC	Overall	2	0.44(0.15,1.27)	1.52	0.129	45.6	0.175
	Caucasian	2	0.44(0.15,1.27)	1.52	0.129	45.6	0.175
	CLL	1	0.28(0.08,0.98)	1.99	**0.047**	–	–
	Multiple Myeloma	1	2.67(0.13,55.98)	0.63	0.526	–	–
CC vs. TC+TT	Overall	2	1.34(0.99,1.82)	1.89	0.059	40.5	0.195
	Caucasian	2	1.34(0.99,1.82)	1.89	0.059	40.5	0.195
	CLL	1	1.64(1.07,2.51)	2.26	**0.024**	–	–
	Multiple Myeloma	1	1.10(0.71,1.69)	0.41	0.682	–	–
TT vs. CC	Overall	2	0.41(0.14,1.19)	1.64	0.100	48.7	0.163
	Caucasian	2	0.41(0.14,1.19)	1.64	0.100	48.7	0.163
	CLL	1	0.26(0.07,0.90)	2.12	**0.034**	–	–
	Multiple Myeloma	1	2.61(0.13,54.77)	0.62	0.536	–	–
TC vs CC	Overall	2	0.77(0.57,1.06)	1.61	0.107	0	0.375
	Caucasian	2	0.77(0.57,1.06)	1.61	0.107	0	0.375
	CLL	1	0.67(0.43,1.05)	1.76	0.078	–	–
	Multiple Myeloma	1	0.89(0.57,1.37)	0.54	0.591	–	–
T vs. C	Overall	2	0.75(0.47,1.18)	1.24	0.215	63.3	0.099
	Caucasian	2	0.75(0.47,1.18)	1.24	0.215	63.3	0.099
	CLL	1	0.60(0.41,0.87)	2.68	**0.007**	–	–
	Multiple Myeloma	1	0.95(0.63.1.43)	0.24	0.812	–	–

OR, odds ratio; CI, confidence interval; CLL, chronic lymphocytic leukemia. Bold type represents significantly difference.

### Sensitivity and publication bias tests

**Figure 3:**
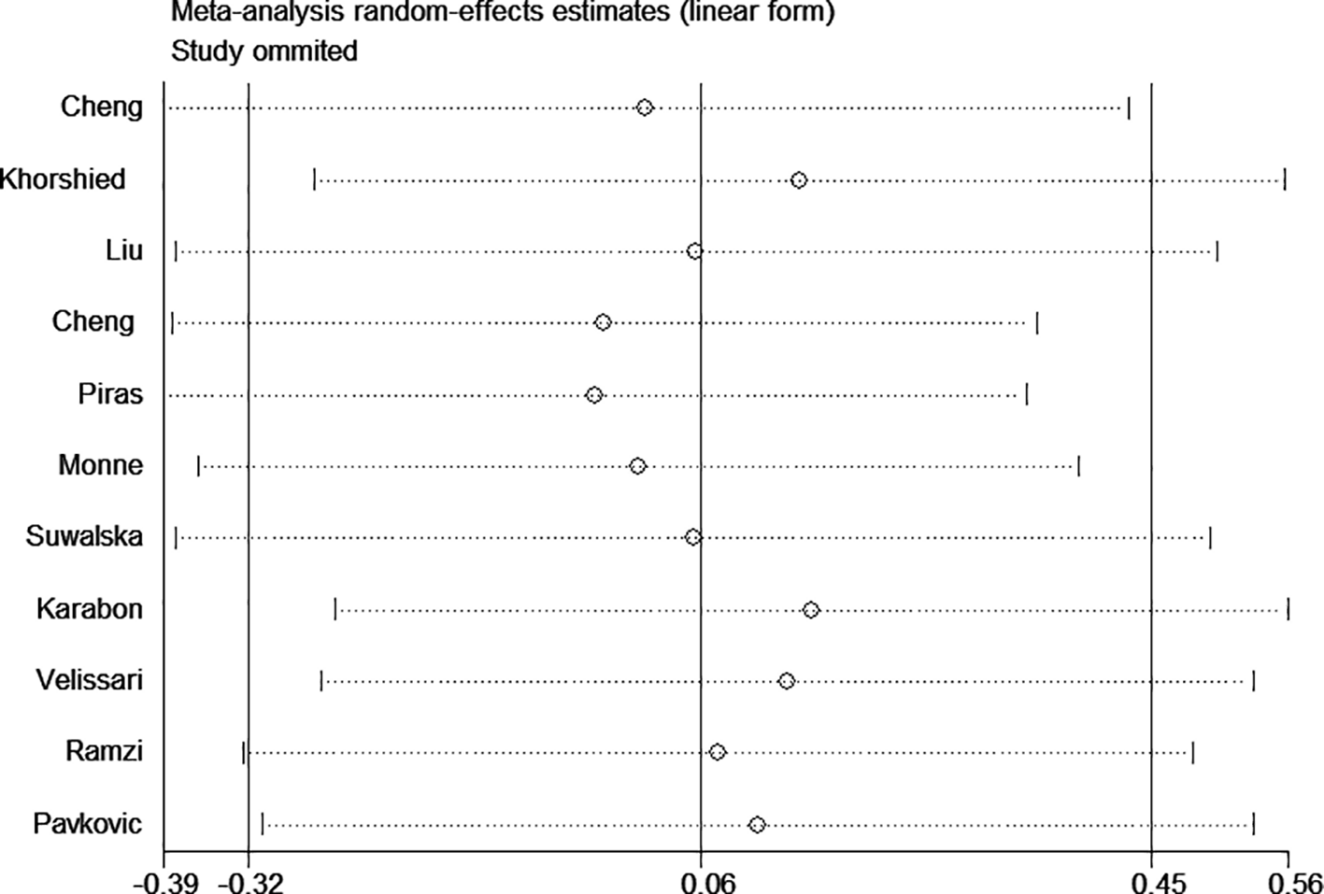
Sensitivity analysis for association between CTLA-4 49A/G polymorphism and hematologic malignancy risk (GG vs. AA).

**Figure 4:**
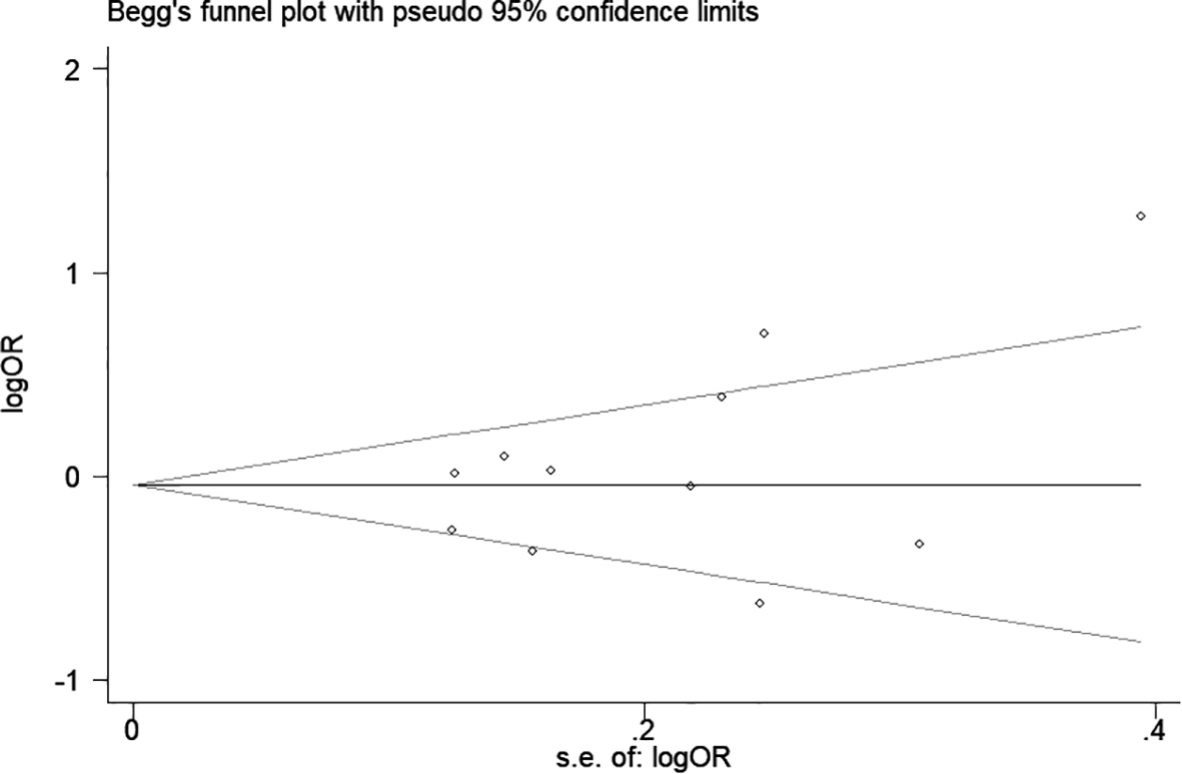
Begg’s funnel plot for association between CTLA-4 49A/G polymorphism and hematologic malignancy risk (G vs. A).

Significant heterogeneity was observed in some comparison models; thus, sensitivity analysis was performed to evaluate the influence of each separate study, and the result indicated the omission of any single study did not significantly change the direction of estimates (**Figure 3**). In addition, there was no publication bias was detected according to Begg rank correlation test (P = 0.312) and Egger linear regression test (P = 0.218) (**Figure 4**).

## Discussion

CTLA-4 is expressed on activated T cells and negatively regulates their activation and proliferation (27). Previous studies have shown that CTLA-4 regulates the duration and intensity of T-cell-mediated immune responses by competitively binding to the costimulatory B7 molecule and activating FAS-dependent cell apoptosis in T cells (27). Recently, abnormal expression of the CTLA-4 gene has been documented in many types of cancers, suggesting that it may contribute to the development and progression of cancer (10, 21, 24). Considering that genetic polymorphisms may affect gene expression and even protein function, the relationship between CTLA-4 gene polymorphisms and different types of malignant diseases has been widely explored. In this meta-analysis, we summarized the results of 13 relevant studies and highlighted the potential relationship between CTLA-4 gene polymorphisms and hematologic malignancies. Our meta-analysis suggested that the CTLA-4 49A/G polymorphism is associated with a decreased NHL, multiple myeloma, and leukemia risk, while the CTLA-4 319C/T polymorphism is significantly associated with a decreased CLL risk.

The difference between lymphoid tumors and other tumors is that this malignant tumor originates from the immune system itself. Most lymphoid tumors originate from mature B and T lymphocytes (28). Therefore, the role of the immune pathway in lymphadenopathy is very complex. As a typical immune regulatory checkpoint, CTLA-4 plays an important role in T-cell anergy and T and B-cell inhibitory responses (27). Therefore, abnormal expression of CTLA-4 may play a role in the pathogenesis of malignant lymphoid tumors. In addition, SNPs in the CTLA-4 gene are widely believed to alter the activity of the promoter and regulate the expression level of CTLA-4. The CTLA4 gene is located on chromosome 2q33 and can exert its negative regulatory effect on T-cell proliferation and activation by competing with CD28 for binding to B7-1/B7-2, downregulating T-cell responses, and peripheral tolerance (29). When CTLA4 is overexpressed, the inhibitory signals produced exceed the immunogenicity provided by tumor cell surface antigens, downregulating or terminating T-cell activation, leading to immune escape of the tumor (30). The abnormal expression of the CTLA-4 gene has been documented in many types of cancers, and it may contribute to the occurrence and progression of cancer (8, 31). Considering that genetic polymorphisms may affect gene expression and even protein function, the relationship between CTLA-4 gene polymorphisms and different types of malignant diseases has been widely explored.

Currently, the pathogenesis of lymphoma is not fully understood, and its occurrence and development involve a multifactorial and multistep process (32). Identifying high-risk populations, clarifying pathogenesis, and increasing treatment options are the most urgent issues to address. Epidemiological studies have shown that the onset of lymphoma is closely related to certain factors. A family history of lymphoma, age, region, race, diet and lifestyle habits, and viral and bacterial infections may all be risk factors for lymphoma (33). Reports also suggest that factors such as tall stature, early immune function, nutritional status, and growth hormone levels play a role in the onset of lymphoma (34). Changes in immune function increase the risk of lymphoma, but the relationship between CTLA4 polymorphisms and susceptibility to hematologic malignancies is inconsistent (21–24). The reasons for these differences are diverse; for example, the origin of the tumor can affect the results of meta-analyses, so we conducted subgroup analyses of CTLA4 polymorphisms by cancer type. The results indicate that the +49A/G polymorphism is associated with an increased risk of NHL, multiple myeloma, and leukemia but not with CLL. In addition, our study results suggest that the 60A/G, 318T/C, and 1661A/G polymorphisms are not associated with hematologic malignancies. However, these results should be interpreted with caution. Due to the inclusion of only 1-2 case-control studies for certain cancer types, the ability to reveal reliable associations may be limited; therefore, further research is needed to validate these associations in the future. In the current meta-analysis, the impact on different racial groups was also analyzed. The +49A/G polymorphism was associated with an increased risk of cancer in Asians but was associated with an decreased cancer risk in Caucasians, while the CTLA-4 319C/T polymorphism was associated with a notable decreased cancer risk in Caucasians. These results strongly suggest that genetic diversity and interactions between genetic variations among different racial groups may lead to different cancer risks. Both racial and environmental factors influence the risk of cancer in different populations. In the future, more research should be conducted to analyze these associations, particularly gene-environment and gene-gene interactions.

Meta-analysis can overcome some of the issues caused by single studies, such as small sample sizes, selection bias, and low-test power; therefore, it is considered a powerful tool for integrating conflicting results from different studies. However, limitations should be noted in our meta-analysis. First, in some subgroups, the number of cases and controls was relatively small, which may have limited the statistical power. Second, significant heterogeneity was observed in some comparisons, which may stem from differences in the histopathological classification of hematologic malignancies and variations in gene detection methods. Third, due to the lack of uniform gene-environment interaction data in the included studies, we were unable to further stratify the data by other factors (such as age, sex, alcohol consumption, smoking status, diet, and other lifestyle factors). Fourth, no available data to examine the association between CTLA-4 polymorphisms and clinical characteristics or comorbidities.

In conclusion, this meta-analysis suggests a significant association between CTLA-4 49A/G and CTLA-4 319C/T with hematologic malignancy risk. However, CTLA-4 60A/G, 318T/C, and 1661A/G polymorphisms showed no significant association with susceptibility to hematologic malignancies. Therefore, further studies with larger sample sizes, different ethnicities, and various types of cancer are needed to confirm these findings.

## Data Availability

The original contributions presented in the study are included in the article/supplementary material. Further inquiries can be directed to the corresponding author.
